# Phenotypic Variation in Infants, Not Adults, Reflects Genotypic Variation among Chimpanzees and Bonobos

**DOI:** 10.1371/journal.pone.0102074

**Published:** 2014-07-11

**Authors:** Naoki Morimoto, Marcia S. Ponce de León, Christoph P. E. Zollikofer

**Affiliations:** 1 Laboratory of Physical Anthropology, Graduate School of Science, Kyoto University, Kyoto, Japan; 2 Anthropological Institute, University of Zurich, Zurich, Switzerland; University of Florence, Italy

## Abstract

Studies comparing phenotypic variation with neutral genetic variation in modern humans have shown that genetic drift is a main factor of evolutionary diversification among populations. The genetic population history of our closest living relatives, the chimpanzees and bonobos, is now equally well documented, but phenotypic variation among these taxa remains relatively unexplored, and phenotype-genotype correlations are not yet documented. Also, while the adult phenotype is typically used as a reference, it remains to be investigated how phenotype-genotye correlations change during development. Here we address these questions by analyzing phenotypic evolutionary and developmental diversification in the species and subspecies of the genus *Pan*. Our analyses focus on the morphology of the femoral diaphysis, which represents a functionally constrained element of the locomotor system. Results show that during infancy phenotypic distances between taxa are largely congruent with non-coding (neutral) genotypic distances. Later during ontogeny, however, phenotypic distances deviate from genotypic distances, mainly as an effect of heterochronic shifts between taxon-specific developmental programs. Early phenotypic differences between *Pan* taxa are thus likely brought about by genetic drift while late differences reflect taxon-specific adaptations.

## Introduction

The ready accessibility of population-wide genotypic and phenotypic data from humans and our closest relatives, the great apes, has spurred a large number of studies investigating the relationship between patterns of genotypic and phenotypic evolution. One central issue is the relative role of neutral versus adaptive evolutionary processes in shaping genotypic and phenotypic variation. A steadily growing number of studies indicates that variation of cranial morphology among modern human populations, and between modern humans and fossil hominins (species related more closely to modern humans than to great apes) largely reflects the effects of genetic drift, while only a small proportion of variation can be attributed to selection [1], [2], [3], [4], [5], [6], [7], [8], [9], [10]. Fossil hominin aDNA now also permits insights into earlier phases of human population and evolutionary history at an unprecedented level of detail [11], [12], [13], [14], [15]. These analyses are limited, however, by the “aDNA preservation horizon”, which is currently around 50,000 years BP for fossil hominin nDNA, and around 400,000 years BP for mtDNA from temperate zones [16].

One possible solution to investigate genotype-phenotype evolution beyond this horizon is to study living great ape species as a model system. The genus *Pan* represents the best model for this purpose, since it is our closest living relative, its species, subspecies and population structure is now genetically well-documented [17], [18], [19], [20], and population history and genetic diversification are well understood [18], [19], [21], [22], [23]. To date, two *Pan* species, *P. troglodytes* (common chimpanzee) and *P. paniscus* (bonobo) are recognized, and *P. troglodytes* is subdivided into four subspecies (*P. t. troglodytes*, *P. t. schweinfurthii*, *P. t. verus* and *P. t. ellioti*) [19]. Also, these *Pan* taxa have been the subject of detailed anatomical [24], [25], [26], [27], [28], morphological [29], [30], [31], [32], [33], phylogeographic [17], [19], [23], [34], and behavioral [32], [35], [36], [37], [38], [39], [40] studies.

The extant *Pan* taxa are closely related to each other, which represents several advantages for comparative analyses. First, genotypic differences between taxa are small compared to variation within each taxon, such that the number of genes associated with phenotypic differentiation during (sub-) speciation is expected to be comparatively small [41]. Second, diversity among *Pan troglodytes* taxa represents patterns of incipient speciation, which are not yet blurred by long-term processes of taxon-specific specialization and/or convergence [42], [43]. Also, we may note that the estimated time frame of *Pan* speciation [19], [23] is comparable to that of our own genus *Homo* (ca. 2 million years).

Despite the increasing knowledge about *Pan* taxa, it still remains to be explored how changes at the level of the genotype are linked to changes at the level of the phenotype during speciation. The first aim of this study is thus to provide new phenotypic data documenting the evolutionary divergence of *Pan* taxa, and to relate this new evidence to the well-established body of genotypic evidence. While evolutionary studies traditionally focus on variation in craniodental features e.g. [44], [45], we study here morphological variation of the femoral shaft ( =  diaphysis). The femur is a functionally highly constrained element of the postcranial skeleton, and can thus be expected to be under strong stabilizing selection.

Most studies exploring genotype-phenotype relationships in great apes and humans have naturally focused on adult morphologies. This is because taxon-specific morphological features are thought to be more clearly expressed in adults than in juveniles. However, there is clear evidence that the phenotypes of early ontogenetic stages, and patterns of developmental change, are highly informative about patterns of evolutionary divergence at the levels of skeletal structure e.g. [46], [47], [48], [49], [50], [51], [52], [53], of locomotor behaviors [35], [37], and of social interactions [54]. The second aim of this study is thus to expand the scope of genotype-phenotype comparisons by taking into account the perspective of ontogeny. Here we explore how genotype-phenotype relationships change during the development of the femoral diaphysis in the different *Pan* taxa, and relate this information to evolutionary change at the level of the genotype and phenotype. Specifically, we explore when during ontogeny the effects of drift versus selection become evident in taxon-specific phenotypes.

Measuring genotype-phenotype relationships is a complex endeavor, both theoretically and practically, and requires several model assumptions. In the standard model of quantitative population genetics, phenotypic variance *V*_P_ is the combination of genetic variance *V*_G_ and environmental variance *V*_E_: *V*_P_ = *V*_E_+*V*_G_. Empirical data and theoretical considerations indicate that, for complex traits, phenotypic variance can be approximated by *V*_P_ = *V*_E_+*V*_A_, where *V*_A_ represents additive genetic variation (the portion of phenotypic variation that can be explained by the cumulative effects of allelic variation) [55]. The question of interest here is how *V*_P_ and *V*_A_ evolve in segregating populations. In a constant environment (*V*_E_ = const.), *V*_P_ = *V*_A_, such that phenotypic variation reflects additive genotypic variation. Under these basic model assumptions, effects of drift and selection are typically estimated by comparing neutral genotypic distances with non-neutral distances [56], [57], [58], [59], [60]. The former distances (*F*_ST_: genetic variation within subpopulation relative to total genetic variation [61], [62]) are estimated from non-coding genetic markers thought to evolve under no selection such as STRs (short tandem repeats) and non-coding SNPs (single nucleotide polymorphisms) [63]. The latter distances are typically estimated from continuous quantitative genetic traits (*Q*_ST_: evaluated in analogy to *F*_ST_ [64]) assuming additive genetic effects [64]. The question is whether *Q*_ST_ is equal to, smaller than, or larger than *F*_ST_, which indicates neutral evolution, uniform or stabilizing selection, and diversifying selection, respectively [65].

*Q*_ST_ can be estimated from phenotypic distance *P*_ST_ [66] using a measure of heritability (*h* ^2^, proportion of additive genetic variance to phenotypic variance, *V*_A_/*V*_P_) [66], [67], [68], [69], [70]. In wild populations, heritability *h* ^2^ is often unknown and needs to be estimated from largely comparable lab studies. Furthermore, *h* ^2^ tends to change due to *in-vivo* environmental effects that accumulate during an individual’s lifetime, and due to developmental changes in gene activation patterns [71], [72], [73]. In any case, estimates of *h* ^2^ affect the distance measures expressed by *Q*_ST_, such that estimating the relative contribution of additive genetic and *in-vivo* environmental effects to *P*_ST_ remains a challenge [74].

A further challenge of *F*_ST_−*Q*_ST_ comparisons is the practical difficulty in measuring genotypic and phenotypic distances. Genotypic distances have been typically calculated using population-specific allele frequencies [75] (e.g., in Nei’s standard distance *D*_a_ [76] and Cavalli-Sforza and Edwards chord distance *D*_CH_ [77]). One problem is that sample sizes of wild populations are often limited, which makes it difficult to estimate population-specific allele frequencies and within-population variation. Complementary methods have thus been proposed, e.g. Principal Components Analysis (PCA) of genetic data [78], [79]. While phenotypic distances have traditionally been evaluated from arrays of linear and angular measurements, geometric morphometrics (GM) offers elegant methods to quantify complex patterns of phenotypic variation [80], [81], [82]. In GM, biological form is typically measured by the spatial configuration (3D geometry) of anatomical points of reference, so-called landmarks [83], [84]. Alternatively, various methods of GM have been developed to quantify the shape of landmark-free biological structures such as outlines [85], endocranial cavities [86] and longbone shafts [46], [87]. One key feature of all GM methods is that phenotypic variation can simultaneously be represented in physical (three-dimensional) space by means of graphical interpolation and in multivariate space by means of PCA. PCA thus provides an ideal means to compare multivariate genotypic and phenotypic data independent of underlying population models.

## Materials and Methods

Volumetric data of the femora of *N* = 146 *Pan* specimens were acquired with computed tomography (CT) (*N* = 50 *Pan troglodytes troglodytes*, *N* = 39 *P.t. schweinfurthii*, *N* = 26 *P. t. verus*, *N* = 31 *P. paniscus*; see Figs. S1 and S2, Table S1, and Text S1 and S2 for details on sample structure). *P. t. troglodytes* and *P. t. verus* specimens were obtained from the collections of the Anthropological Institute and Museum of the University of Zurich (AIMUZH), *P. t. schweinfurthii* specimens were obtained from the collections of the Royal Africa Museum, Tervuren, Belgium (MRA), and *P. paniscus* specimens were obtained from AIMUZH and MRA (Table S1). Each taxon is represented by four consecutive ontogenetic stages from infancy to adulthood. These were defined according to dental eruption: m2 (second deciduous molar erupted), M1, M2, M3 (first, second, third permanent molars erupted). In *Pan*, m2, M1, M2 and M3 erupt approximately at 0.5–0.83, 3, 7 and 11 years after birth, respectively [88].

Because femoral epiphyses are not yet ossified during the early stages of ontogeny, we focus on diaphyseal morphology. Effects of *in-vivo* bone modification in the femur have been studied in various *Pan* taxa, and it has been shown that ontogenetic changes in femoral morphology reflect an underlying developmental program that is fairly independent of environmental influences [87]. In other words, environmental variance *V*_E_ remains approximately constant throughout ontogeny [31], [87], [89] (see Text S3), which is an important prerequisite to estimate *Q*_ST_ from *P*_ST_ [74].

**Figure 1:**
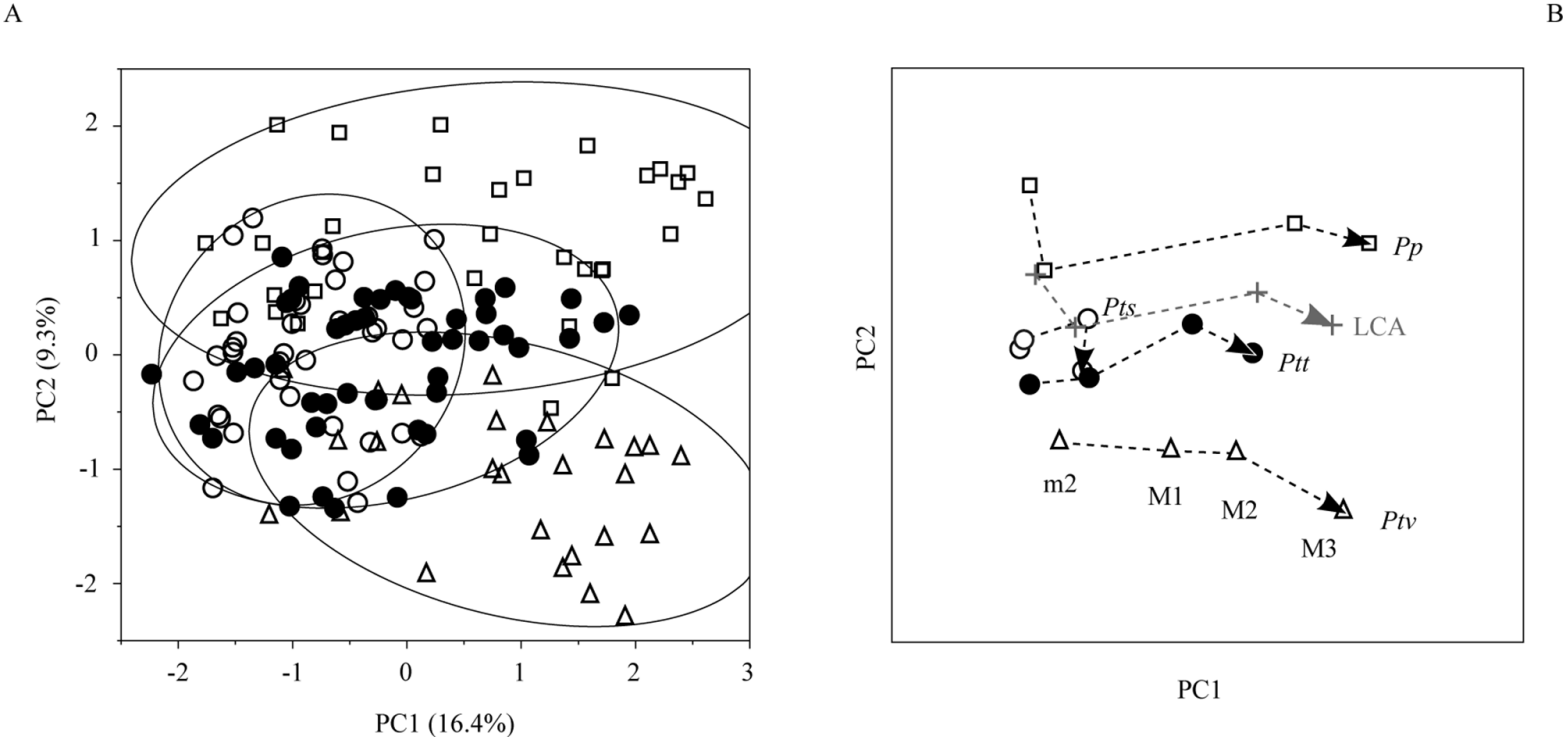
Femoral diaphyseal shape variation in an ontogenetic sample of *Pan* taxa. A: Variation along the first two principal components of shape, PC1 and PC2 (filled circles: *P.t. troglodytes*, open circles: *P.t. schweinfurthii*, open triangles: *P.t. verus*, open squares: *P. paniscus*). Solid outlines show 95%-density ellipses for each taxon. B: plot of mean shapes at consecutive ontogenetic stages. m2: second deciduous molar erupted; M1/M2/M3: permanent molars 1/2/3 erupted. Gray symbols and dashed line indicate the inferred shape at each ontogenetic stage and ontogenetic trajectory of the last common ancestor.

**Figure 2:**
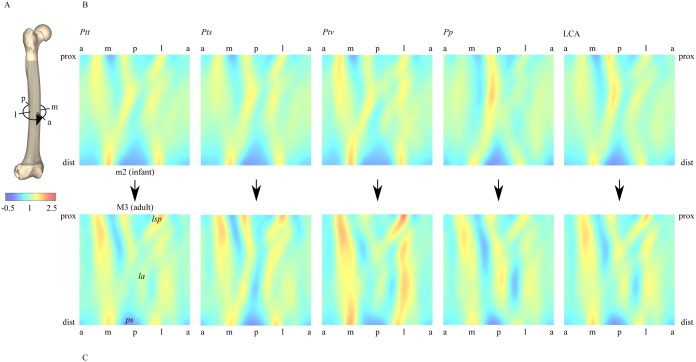
Taxon-specific femoral diaphyseal shapes. A: principle of morphometric map generation (anterior [0°] → medial [90°] → posterior [180°] → lateral [270°] → anterior [360°]). B, C: morphometric maps of taxon-specific morphologies at ontogenetic stages m2 (B, infant) and M3 (C, adult) (false-color images of external surface curvature [relative units]). *la*: linea aspera, *lsp*: lateral spiral pilaster, *ps*: popliteal surface.

To quantify a specimen’s diaphyseal surface morphology the transverse radius of curvature was evaluated for each point of the external (subperiosteal) surface, as specified in ref. [87]. The data of all specimens were then analyzed by means of morphometric mapping (MM) methods [87], [90] (Fig. S3 and Text S1). MM is a landmark-free geometric morphometric method that permits dense sampling of data from smooth surfaces. It is thus well suited to quantify even subtle morphological differences in femoral shaft form between different taxa and/or developmental stages [87], [91], [92], [93]. To correct for size differences between specimens, size is normalized by diaphyseal length and the median value of the radius of curvature. Shape variation is then decomposed into statistically independent shape components, which represent multivariate descriptors of the total femoral diaphyseal morphology. Since MM establishes a direct link between femoral geometry and its multivariate representation, patterns of inter- and intra-group variation can be visualized in multivariate shape space (“morphospace”; Fig. 1) as well as in real (physical) space (Fig. 2). To infer the femoral diaphyseal morphology and its developmental pattern in the last common ancestor (LCA) of *Pan* taxa, the phylogenetic tree of *Pan* taxa was projected onto the morphospace using a model of squared-change parsimony under a Brownian motion model [94] for each ontogenetic stage (Fig. S4) using the software package MorphoJ [95]. Also, MM was used to infer the infant and adult femoral diaphyseal morphology of the LCA (Fig. 2).

Mean femoral diaphyseal shape was calculated for each taxon at each ontogenetic stage *i*, and inter-taxon phenotypic (*i.e.*, morphometric) distance matrices **M** *_i_* were calculated for each stage. As a phenotypic distance metric, the Euclidean distance in morphospace was used. Between-taxon quantitative genetic differentiation (*Q*_ST_) was also estimated for each ontogenetic stage. To this end, pairwise *Q*_ST_s were evaluated from *P*_ST_s with the software RMET 5.0 [96], [97], using PC scores (PC1–3) and a standard estimation of heritability *h* ^2^ = 0.55. This procedure resulted in stage-specific distance matrices **Q** *_i_*.

Genotypic distances between *Pan* taxa (matrices **F**) were calculated from sequence datasets. The sequence data of 150,000 bp on 15 non-coding autosomal regions in *N* = 74 *Pan* specimens were obtained from GenBank (accession number: JF725992–727161 [22]). Inter-taxon genotypic distances were evaluated with various methods; Nei’s standard distance *D*_a_ [76], Cavalli-Sforza and Edwards chord distance *D*_CH_ [77], and Euclidean distances in Patterson’s PC space *D*_PPC_ [78], [79]. Further, *F*_ST_ and *R*_ST_ from published sources were also used to construct genotypic distance matrices ([18], [19], [21], [22]; refs. [18] and [19] use the same marker set) (Table S2).

Overall, three kinds of between-taxon distance matrices **F** (genotypic), **M** (phenotypic) and **Q** (quantitative genetic) were evaluated, and these matrices were used for **F**−**M** and **F**−**Q** (*F*_ST_ − *Q*_ST_ [*P*_ST_]) comparisons. The similarity between these distance matrices was evaluated with principal coordinate analysis (PCO), and assessed statistically with the Mantel test and resampling statistics (see Text S1 and Fig. S3 for details on PCO and resampling statistics). In brief, PCO transforms a between-taxon distance matrix into a “taxon constellation” (i.e., locations of taxa relative to each other in multivariate space). To assess the coincidence between genotypic and phenotypic taxon constellations, we used Procrustes analysis. This method superimposes two or more different constellations using a least-squares criterion. The Mantel test was performed using Relethford’s MANTEL 3.1 (software programs RMET and MANTEL are available at http://employees.oneonta.edu/relethjh/programs/).

The fact that more than two *Pan* taxa are studied here facilitates rather than complicates *F*_ST_–*Q*_ST_ comparisons. For *K* = 2 groups (populations or taxa), one *F*_ST_ distance is compared with one *Q*_ST_ distance. These need to be scaled appropriately with an estimate of *h* ^2^ to permit significant implications on neutral versus adaptive evolution, but *h* ^2^ is typically unknown. For *K*>2 groups (this study: *K* = 4), the structures of two *K*×*K* distance matrices (*F* and *Q*) are compared, and scaling issues can be addressed with methods of matrix-matrix correlation and multidimensional scaling (MDS) such as the PCO method used here e.g. [2], [7], [98], [99], [100]. Assuming that *h* ^2^(*i*) = const. for all groups at a given ontogenetic stage *i*, MDS will thus scale *P*_ST_ and *Q*_ST_ relative to *F*_ST_ even without explicit estimates of *h* ^2^(*i*) (refs. [10], [101]).

These matrix-matrix comparisons permit to assess whether the structure of a phenotypic (**M**) or quantitative-genetic (**Q**) distance matrix is similar to, or deviates from, a putatively neutral genotypic distance matrix **F**. Similarity would imply that **M** and **Q** are scaled versions of **F** (scaling factor *h* ^2^). An important assumption is that the genetic markers to estimate *F*_ST_ follow neutral evolution. This is critical to evaluate the relative role of neutral and adaptive processes from phenotypic data. The genetic markers used here to estimate *F*_ST_ represent non-coding regions [18], [19], [21], [22], so it is reasonable to assume that variation reflects neutral processes.

## Results

Fig. 1 shows commonalities and differences in femoral diaphyseal shape and shape variation between *Pan* taxa. The first two principal components represented here (PC1 and PC2) account for 25.7% of the total shape variation in the sample. There is substantial overlap between taxon-specific distributions of *P. t. troglodytes* and *P. t. schweinfurthii*, but almost no overlap between *P. paniscus* and *P. t. verus* (Fig. 1A). At each ontogenetic stage, taxon-specific mean shapes are statistically different from each other (Fig. 1B, Table S3). Furthermore, taxon-specific ontogenetic trajectories (see SI and refs. [102], [103]) have statistically similar directions through morphospace (Fig. 1B and Table S4). Trajectories differ from each other, however, in their length (mostly along PC1), and in their location in morphospace (mostly along PC2) (Fig. 1B). Trajectories of *P. t. troglodytes* and *P. t. schweinfurthii* are in close vicinity, but the trajectory of the latter taxon is significantly shorter than that of the former. Compared to these taxa, the trajectory of *P. paniscus* is significantly longer (Fig. 1B, Table S5).

Differences between trajectories are already present at the m2 (infant) stage, indicating that taxon-specific femoral shape is established early during ontogeny. The differences in trajectory length indicate that the shape differences between *Pan* taxa increase toward adulthood. Longer trajectories indicate a larger total amount of femoral shape change during ontogeny, and possibly higher rates of shape change. Fig. 2 visualizes the corresponding real-space patterns of femoral diaphyseal shape change from infant to adult for each taxon. Each stage- and taxon-specific diaphyseal shape is represented here with a morphometric map (MM), which represents surface structures around (x-axis) and along (y-axis) the femoral diaphysis. MMs visually confirm that taxon-specific femoral shape is present already at the m2 (infant) stage, and that taxon-specific features become more pronounced toward the M3 (adult) stage.

Using methods of squared-change parsimony [94], it is possible to infer the ontogenetic trajectory of the LCA of *Pan* taxa. The LCA trajectory lies between the trajectory of *P. paniscus* and the average trajectory of *P. troglodytes* taxa (Figs. 1, 2, S4). The length of the LCA trajectory is comparable to that of *P. t. troglodytes*, *P. t. verus*, and *P. paniscus*, but is longer than that of *P. t. schweinfurthii*.

All measures of genotypic distances (*F*_ST_, *D*_a_, *D*_CH_, *D*_PPC_) are highly correlated with each other (Table S6; Mantel test). Genotypic distances (*F*_ST_ and *R*_ST_) evaluated from different marker sets [18], [19], [21], [22] (Table S2) are also concordant with each other (Fig. S5), indicating that potential noise due to the small sample sizes of these studies does not greatly affect the results [104]. In all further comparative analyses we use *D*_PPC_ because evaluation of this distance measure does not presuppose estimation of within-group variance.

**Figure 3:**
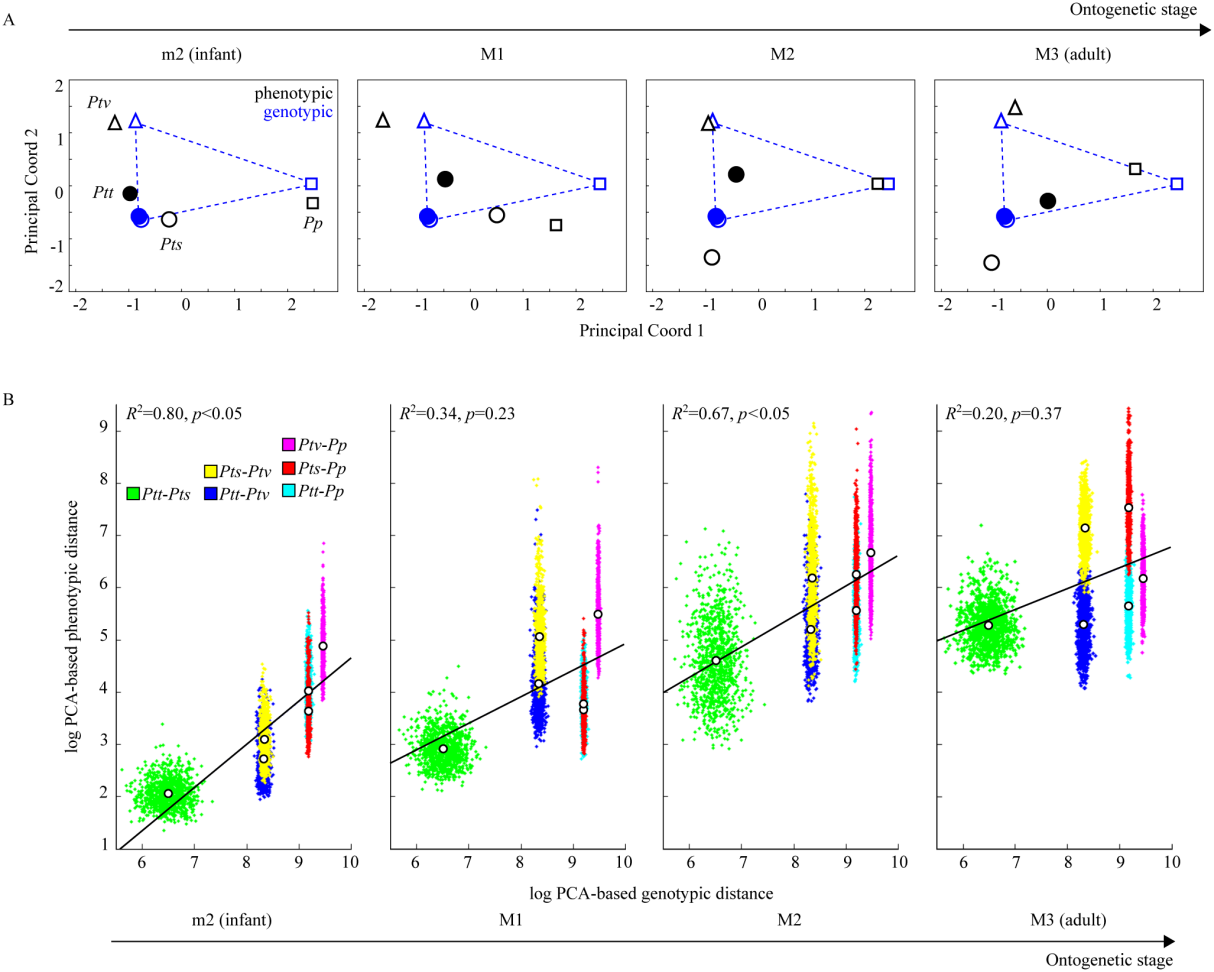
Comparison of genotypic and phenotypic distances between *Pan* taxa. A: Principal Coordinates Analysis (PCO) permits representation of genotypic and phenotypic distance data in the same multivariate space. The four subgraphs show phenotypic data (black dots) for consecutive ontogenetic stages m2, M1, M2, and M3, and genotypic data (same blue dots for all stages). For graphical clarity genotypic data points, which are independent of ontogenetic stage, are connected with dashed lines. Note that during ontogeny the phenotypic distance configuration departs from the neutral genetic distance configuration (see also Fig. S5). B: Correlation between phenotypic and neutral genetic distances between taxa. Each point cloud consists of 1000 randomly sampled phenotypic and genotypic distances between individuals belonging to different *Pan* taxa (resampling procedures are explained in Text S1). Correlation of phenotypic and neutral genetic distances is highest at the m2 (infant) stage and declines towards adulthood (M3). Genetic and phenotypic distances are normalized by their respective median values. Note overall increase of phenotypic distance between taxa toward adulthood.

To assess the congruence between genotypic and phenotypic distance matrices, we projected the genotypic and phenotypic PCO data into the same multidimensional space and aligned them with Procrustes Analysis. Patterns of phenotypic similarity among *Pan* taxa (*P*_ST_) are overall congruent with patterns of genetic similarity (*D*_PPC_, *F*_ST_) (Figs. 3A, S5, Tables 1, S6, S7). Figs. 3A and S5 show that the match between genotypic and phenotypic data is closest at the m2 (infant) stage (Table 1; *p*<0.05, Mantel test). While taxa advance along their ontogenetic trajectories, patterns of phenotypic variation tend to deviate from the pattern of genetic variation (Fig. 3A, S5). These results are statistically supported by a resampling test (Fig. 3B). **F**–**M** correlation is highest at the m2 (infant) stage (*R* ^2^ = 0.80, *p* = 0.02), and is lowest at the M3 (adult) stage (*R* ^2^ = 0.20, *p* = 0.37). Likewise, the **F**–**M** correlation between genotypic and phenotypic distances evaluated by a Mantel test is highest at the m2 stage (Tables 1 and S7). **F**–**M** correlation is also significant at the M2 stage, but to a lesser extent than at the m2 stage. The decline in **F**–**M** correlation from infancy to adulthood thus follows a non-monotonous pattern.

**Table 1:** Correlation between genotypic and genotypic distance matrices.

		m2 (infant)	M1	M2	M3 (adult)
genotypic distance^1^−phenotypic distance^2^ (Mantel^3^)	*R* 2	**0.84**	0.15	**0.64**	0.18
	*p*	**<0.01**	0.2609	**<0.01**	0.3478
genotypic distance−phenotypic distance (resampling^4^)	*R* 2	**0.80**	0.34	**0.67**	0.20
	*p*	**0.015**	0.23	**0.045**	0.37
*F*_ST_−*Q*_ST_ test^5^ (Mantel)	*R* 2	**0.72**	0.40	**0.67**	0.10
	*p*	**<0.01**	0.087	**<0.01**	0. 3478

1 Euclidean distance in Patterson’s PC space.

2 Euclidean distance in morphospace (shape PCs).

3 correlation (*R* ^2^) and significance levels (*p*) evaluated with Mantel test (1000 permutations).

4 evaluated with resampling statistics (see methods; Fig. S3C).

5 estimate of heritability *h* ^2^: 0.55.

The results of **F**−**Q** comparisons (i.e., standard *F*_ST_−*Q*_ST_ tests) are similar to the results obtained with PCA/PCO analyses (Table 1). The correlation between *F*_ST_ and *Q*_ST_ [*P*_ST_] is highest at the m2 (infant) stage (*R* ^2^ = 0.72, *p*<0.01), and lowest at the M3 (adult) stage (*R* ^2^ = 0.10, *p* = 0.35). The finding that correlation between genotypic and phenotypic markers decreases during ontogeny is thus independent of the method of genotypic and phenotypic distance measurement.

## Discussion

Investigating the evolutionary divergence between populations and/or closely related taxa at the level of genes and phenes, and inferring underlying processes of selection and drift, has become an important research topic in primatology and anthropology [1], [2], [3], [4], [5], [6], [7], [8], [9], [10]. Progress in this field is fostered by the availability of ever-increasing volumes of genomic and phenomic data, and sophisticated analytical tools to compare patterns of genotypic and phenotypic variation. While DNA sequence data provide *static* structural information about the genome, data at any level above the DNA (from the transcriptome to morphology) provide *dynamic* structural information about the phenotype, which changes during ontogeny. Interestingly, the effect of ontogenetic time on correlations between genotypic and phenotypic variation is still relatively unexplored. For example, ontogenetic time does not appear as an explicit variable in the standard equations relating *V*_P_ to *V*_A_, nor is it typically considered explicitly in *F*_ST_−*Q*_ST_ comparisons.

To fill this gap, we studied femoral diaphyseal shape change in the genus *Pan* and compared patterns of phenotypic divergence (both during development and evolution) with patterns of genotypic divergence. The results presented here yield several new insights into evolutionary and developmental links between genotypic and phenotypic diversification in *Pan*. Before any general inferences can be drawn, it should be reminded, however, that the genotypic and phenotypic data sets studied here represent subsets of the total genotypic/phenotypic evidence that is potentially available for such studies.

The close correspondence between genotypic and phenotypic distances at the earliest ontogenetic stage analyzed here (the m2 stage) gives rise to two alternative hypotheses; H0: if the molecular markers of refs. [18], [19], [21], [22] track neutral evolution then the observed pattern of phenotypic evolution is “neutral-like” within the constraints imposed by stabilizing selection (often described as “wandering around an adaptive optimum” [105], [106], [107]); H1: if the pattern of phenotypic distances between taxa is the result of selection and adaptation, then the molecular markers are non-neutral and carry an adaptive signal. Given the good evidence for neutrality in the molecular markers [108] used here, hypothesis H1 is less likely. Also, the congruence of the genotypic distance patterns evaluated from different marker types (Fig. S5) suggests that H1 is less likely, since one would expect that selection acts differently on different marker types. Our data thus support hypothesis H0, which implies that morphological variation of the femoral diaphysis in infant *Pan* reflects neutral evolutionary diversification between taxa rather than taxon-specific adaptation.

While phenotypic distances between *Pan* taxa at the m2 stage are in good concordance with genotypic distances (*R* ^2^ = 0.8; Fig. 3B), correlations are lower at later ontogenetic stages, and reach a value of *R* ^2^ = 0.2 at adulthood (Figs. 3, S5; Table 1). As already reported in earlier studies [74], [109], [110], correlations between molecular and phenotypic markers are typically low, and this has been interpreted in two ways: (1) that (non-coding) molecular marker variation does not adequately represent the quantitative genetic variation of coding genes that becomes manifest in the phenotype, and (2) that environmental variation has a significant influence on *V*_P_, and hence on *Q*_ST_.

The ontogenetic data presented in this study provide an empirical basis to test these hypotheses. The high correlation (*R* ^2^ = 0.80) between inter-taxon molecular and phenotypic variation at the m2 stage (Fig. 3B) indicates that, during early ontogeny, molecular marker variation indeed represents quantitative genetic variation. Departure from genotypic-phenotypic correspondence during later ontogenetic stages might indicate *in-vivo* modification of the femoral shaft morphology, indicating an increasing contribution of *V*_E_ to *V*_P_ over ontogenetic time. Given the evidence from earlier studies investigating *in-vivo* effects on femoral shaft morphology [31], [87], [89], [111], however, this interpretation is unlikely, and *V*_E_ remains fairly constant from infancy to adulthood [87]. Another possible explanation is size allometry, implying that the observed pattern of phenotypic divergence reflects differences in adult body mass among *Pan* taxa. Since direct data on body mass are available for only few specimens in this study, we use the taxon-specific body masses reported in the literature [112] to test this hypothesis. Taxon-specific means of PC scores at adulthood are not correlated with adult body masses of *Pan* taxa (Fig. S6, Table S8). It is thus unlikely that the observed pattern of divergence is due to allometry.

After excluding major environmental and allometric effects, it appears most likely that phenotypic divergence is caused by genetically determined taxon-specific developmental programs. This implies that the genetic variance *V*_G_ changes during ontogenetic time *t*: *V*_P_(*t*) = *V*_E_+*V*_G_(*t*). In the present case, it is not known whether *V*_G_(*t*) can be approximated by additive genetic variance *V*_A_(*t*) alone, or whether non-additive effects have to be taken into account. Several alternative hypotheses must thus be considered to explain the observed pattern of phenotypic divergence. Under the additive genetic variance model [*V*_P_(*t*) = *V*_E_+*V*_A_(*t*)], our hypothesis is that the genes mediating early ontogeny (up to the m2 stage) evolved by neutral processes (*Q*_ST_ ∼*F*_ST_), whereas the genes mediating late ontogeny (from m2 to adulthood) evolved under selection (*Q*_ST_>*F*_ST_), probably as an adaptation to taxon-specific locomotor regimes. An alternative hypothesis is that non-additive effects *V*_N_ are a function of developmental time: *V*_P_(*t*) = *V*_E_+*V*_A_(*t*)+*V*_N_(*t*). With the currently available empirical evidence, we cannot decide between these hypotheses. In any case, the molecular markers used here to estimate *V*_G_ are unlikely to represent variation in the actual coding genes that cause *V*_P_ to increase over ontogenetic time [110].

In spite of these uncertainties, our data permit inferences on the developmental mechanisms that cause taxon-specific differences in femoral diaphyseal shape, and to speculate on their genetic basis. As shown in Fig. 1B, taxon-specific ontogenetic trajectories set out at similar locations along PC1, but differ in their length. This pattern indicates differences in taxon-specific *rates* of development from the m2 stage onward, resulting in significant differences between adult morphologies. Evolutionary divergence via differential developmental rates is well-known as heterochrony. It thus appears that heterochronic shifts played a major role in the development of the adult femoral morphologies of *Pan* taxa. Such shifts might be effected by changes in a small number of developmental genes [113], [114], which are difficult to trace with standard molecular markers, but might be further investigated with whole-genome comparisons [23].

It has been shown that a marked paedomorphic pattern is expressed in the skull relative to the postcranial skeleton in bonobos (*P. paniscus*) compared to common chimpanzees (*P. troglodytes*) [33], [115], [116]. The present study shows that the femur also exhibits heterochronic variation among *Pan* taxa. It is interesting to note that the femoral diaphysis of bonobos exhibits peramorphic development compared to common chimpanzees. This mosaic structure of evolutionary developmental modification is in concordance with the observation made earlier that *P. paniscus* is not just a paedomorphic chimpanzee [116], [117]. It remains to be elucidated whether cranial and postcranial ontogenies are governed by the same set of “heterochrony genes”, which have different local effects, or whether different sets of heterochrony genes are expressed locally [113], [118].

Currently, we can only speculate about the adaptive significance of taxon-specific heterochronic modifications of femoral development, since more comparative field data are necessary to specify the diversity of locomotor behaviors and their ontogeny in all *Pan* taxa. The inferred femoral diaphyseal morphology and developmental trajectory of the *Pan* LCA indicates that the peramorphic pattern as in *P. paniscus*, *P. t. troglodytes* and *P. t. verus* represents the primitive state whereas the paedomorphic (rate hypomorphic) pattern as in *P. t. schweinfurthii* represents the derived state. The inferred femoral diaphyseal morphology of the LCA at the adult stage is relatively close to the morphology of adult *P. paniscus* and *P. t. troglodytes*. The locomotor repertoire of the LCA might thus have been close to that of adult *P. paniscus* and *P. t. troglodytes*.

The data presented here provide empirical insights into the role of neutral and adaptive evolutionary mechanisms at the level of genes and phenes. In the system studied here, it appears that – among the closely related *Pan* taxa – early developmental genes evolve mostly neutrally and produce neutral taxon-specific phenotypes, while selection acts on late developmental genes (most likely on those involved in the regulation of developmental rates) and produces adaptive phenotypes.

Evidence for this pattern of evolution has also been found in the hominin clade. For example, the pattern of genotypic and phenotypic divergence between *Homo sapiens* and *H. neanderthalensis* is concordant with a model of neutral evolution by mutation and drift [6], [8]. Also, parallel ontogenetic trajectories and heterochronic divergence during late ontogeny are reported for *Homo sapiens* and *H. neanderthalensis* [51]. Likewise, it appears that genetic and phenotypic divergence in early *Homo* and between modern human populations is governed to a large extent by neutral processes [1], [3], [5], [10], [119], [120]. Our data indicate that this pattern of evolution might be more general than currently thought and characteristic not only for *Homo* but also for the taxa descending from the last common ancestor of humans and chimpanzees. It remains to be tested whether the observed patterns of developmental diversification in *Pan* also characterize the developmental diversification in other great ape taxa.

As a general outcome of this study, we may state that the phenotype of early developmental stages conveys a better neutral phylogenetic signal than the adult phenotype. This finding is in contrast with the traditional notion that the fully-developed adult phenotype is most significant for taxonomy and phyletic inference. The close match between patterns of neutral molecular and phenotypic variation during early ontogeny, however, indicates that immature individuals are of special relevance to infer phylogenetic relationships, although taxon-specific features are less expressed in early stages of ontogeny (Fig. 2B) compared to late stages (Fig. 2C). Femoral diaphyseal morphology of hominoids provides a good example. While adult-based studies often show similarities of femoral diaphyseal morphology among great apes to the exclusion of humans e.g. [121], [122], [123], at an early developmental stage humans and chimpanzees are grouped together to the exclusion of gorillas [46]. Furthermore, our data may explain why previous meta-analyses showed a generally low correlation of *F*_ST_ and *Q*_ST_ in adult phenotypes [74], [110], [124]. Generalizing our findings to hominoid (and hominin) evolution, the comparison of immature and adult phenotypes will permit a better discrimination between phyletic and adaptive signals in the phenotype.

## Supporting Information

Figure S1 **Geographical distribution and taxonomy of** ***Pan*** **(modified from ref.** [22] **).**
(TIF)

Figure S2 **Sample structure by taxon and age class.** A, distribution of femoral diaphyseal length (measured as the linear distance between proximal and distal epiphyseal lines). B: distribution of femoral diaphyseal cross-sectional area (measured as the median of cross-sectional areas between proximal and distal epiphyses). Filled circles: *P.t. troglodytes*, open circles: *P.t. schweinfurthii*, open triangles: *P.t. verus*, open squares: *P. paniscus*. Age classes: m2: second deciduous molar erupted; M1/M2/M3: permanent molars 1/2/3 erupted. Each symbol represents a specimen; black lines/whiskers indicate mean and range; red boxes and whiskers indicate first/third quartiles and median.
(TIF)

Figure S3 **Principle of morphometric mapping.** A, 3D representation of the right femur. B, principle of cylindrical projection (anterior [0°] → medial [90°] → posterior [180°] → lateral [270°] → anterior [0°]).
(TIF)

Figure S4 **Phylogenetic tree in morphospace.** The phylogenetic tree (blue lines; diamonds indicate the inferred state of last common ancestor at each ontogenetic stage) of the genus *Pan* is projected onto the shape space using a model of squared-change parsimony. A: m2 (infant), B: M1, C: M2, D: M3 (adult) stage. Gray symbols and line indicate the inferred ontogenetic trajectory of the last common ancestor.
(TIF)

Figure S5 **Phenetic and genetic similarity between** ***Pan*** **taxa.** Principal Coordinates Analysis (PCO) of phenetic and genetic distance data. Phenetic data (black) are given for consecutive ontogenetic stages (connected with dashed lines). Genetic data (color) are from ref. [18] (blue), ref. [19] (green), ref. [21] (red), and ref. [22] (magenta). Note that during ontogeny the phenetic distance configuration departs from the genetic distance configuration.
(TIF)

Figure S6 **Correlation of taxon-specific means of adult body weight and PC scores.** Taxon-specific means of adult body weight was calculated as a mean of male and female body weight taken from the literature [112].
(TIF)

Table S1 **Specimen list.** The following specimens are used in this study. AIMUZH: Anthropological Institute and Museum of University of Zurich. MRA: Royal Africa Museum, Tervuren, Belgium.
(DOCX)

Table S2 **Genetic distances between** ***Pan*** **taxa (** ***F*** **_ST_ and** ***R*** **_ST_).**
(DOCX)

Table S3 **Phenetic distances between taxon-specific mean shapes.**
(DOCX)

Table S4 **Divergence of ontogenetic vector.**
(DOCX)

Table S5 **F-test on taxon-specific variance along PC1.**
(DOCX)

Table S6 **Correlation of genetic and phenetic distances.**
(DOCX)

Table S7 **Correlation between phenetic and genetic distance matrices.**
(DOCX)

Table S8 **Correlation between PC scores and taxon-specific adult body masses.**
(DOCX)

Text S1 **Materials and methods.**
(DOCX)

Text S2 **Habitats of** ***Pan*** **taxa.**
(DOCX)

Text S3 ***In-vivo*** **bone modification in the femur of** ***Pan*** **taxa.**
(DOCX)
