# Supplementary figures and images for: Phenotypic Variation in Infants, Not Adults, Reflects Genotypic Variation among Chimpanzees and Bonobos
Source: PLoS One. 2014 Jul 11;9(7):e102074. doi: 10.1371/journal.pone.0102074 (PMC4094530; doi:10.1371/journal.pone.0102074)

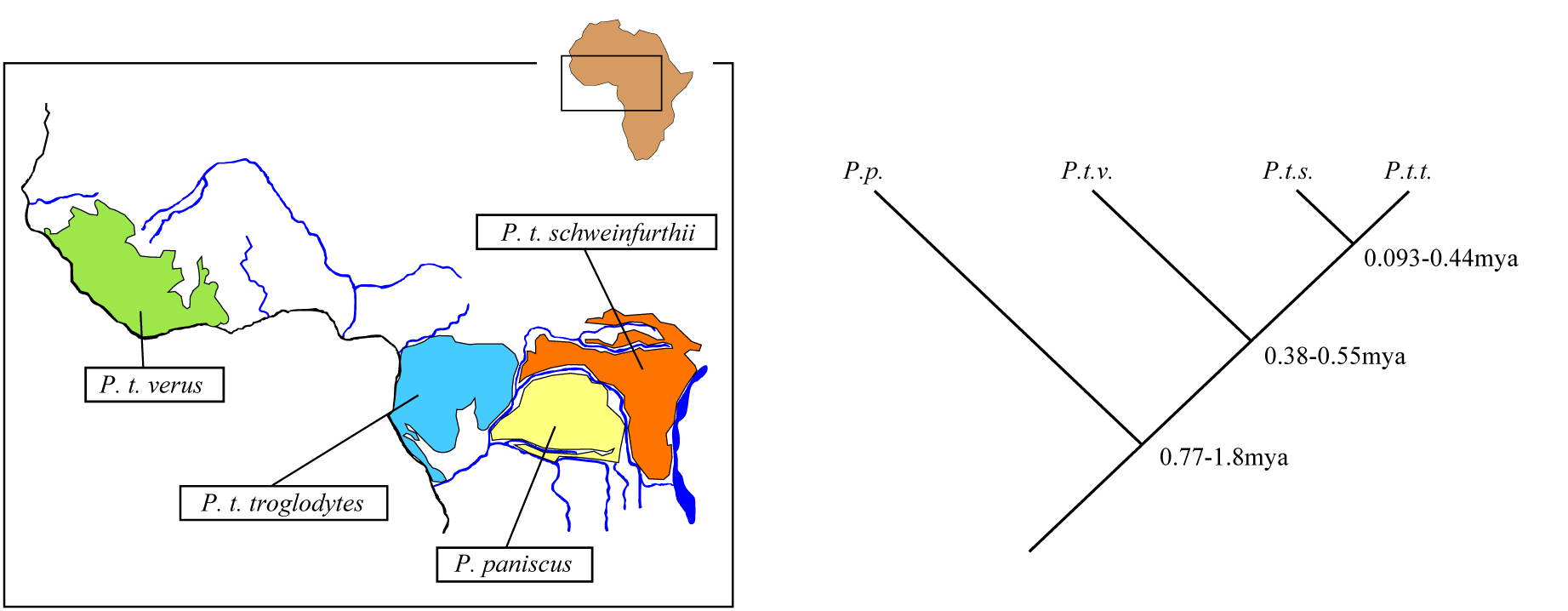

Supplement: Figure S1 — Geographical distribution and taxonomy of Pan (modified from ref. [22] ). (TIF) [file pone.0102074.s001.tif]

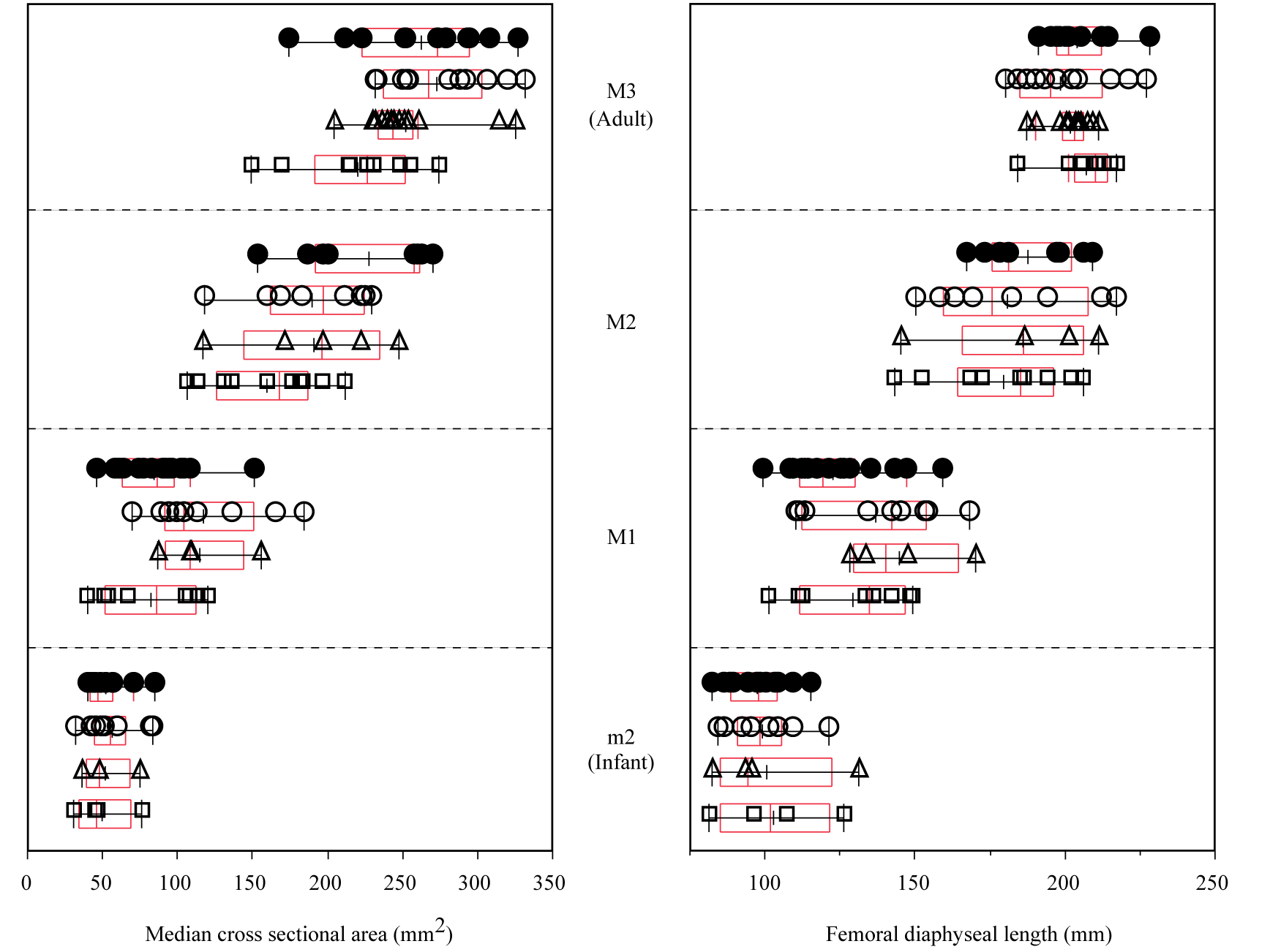

Supplement: Figure S2 — Sample structure by taxon and age class. A, distribution of femoral diaphyseal length (measured as the linear distance between proximal and distal epiphyseal lines). B: distribution of femoral diaphyseal cross-sectional area (measured as the median of cross-sectional areas between proximal and distal epiphyses). Filled circles: P.t. troglodytes, open circles: P.t. schweinfurthii, open triangles: P.t. verus, open squares: P. paniscus. Age classes: m2: second deciduous molar erupted; M1/M2/M3: permanent molars 1/2/3 erupted. Each symbol represents a specimen; black lines/whiskers indicate mean and range; red boxes and whiskers indicate first/third quartiles and median. (TIF) [file pone.0102074.s002.tif]

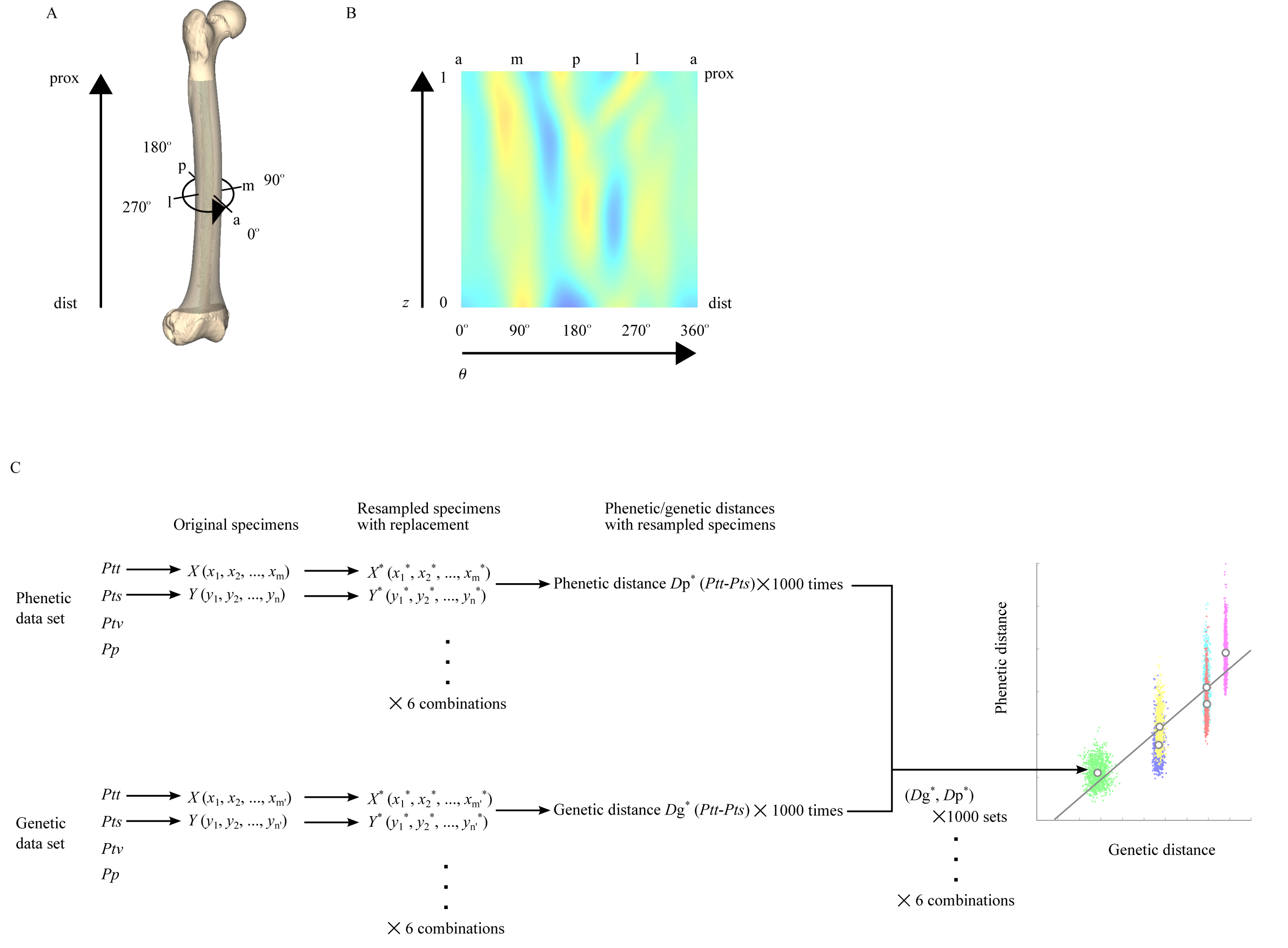

Supplement: Figure S3 — Principle of morphometric mapping. A, 3D representation of the right femur. B, principle of cylindrical projection (anterior [0°] → medial [90°] → posterior [180°] → lateral [270°] → anterior [0°]). (TIF) [file pone.0102074.s003.tif]

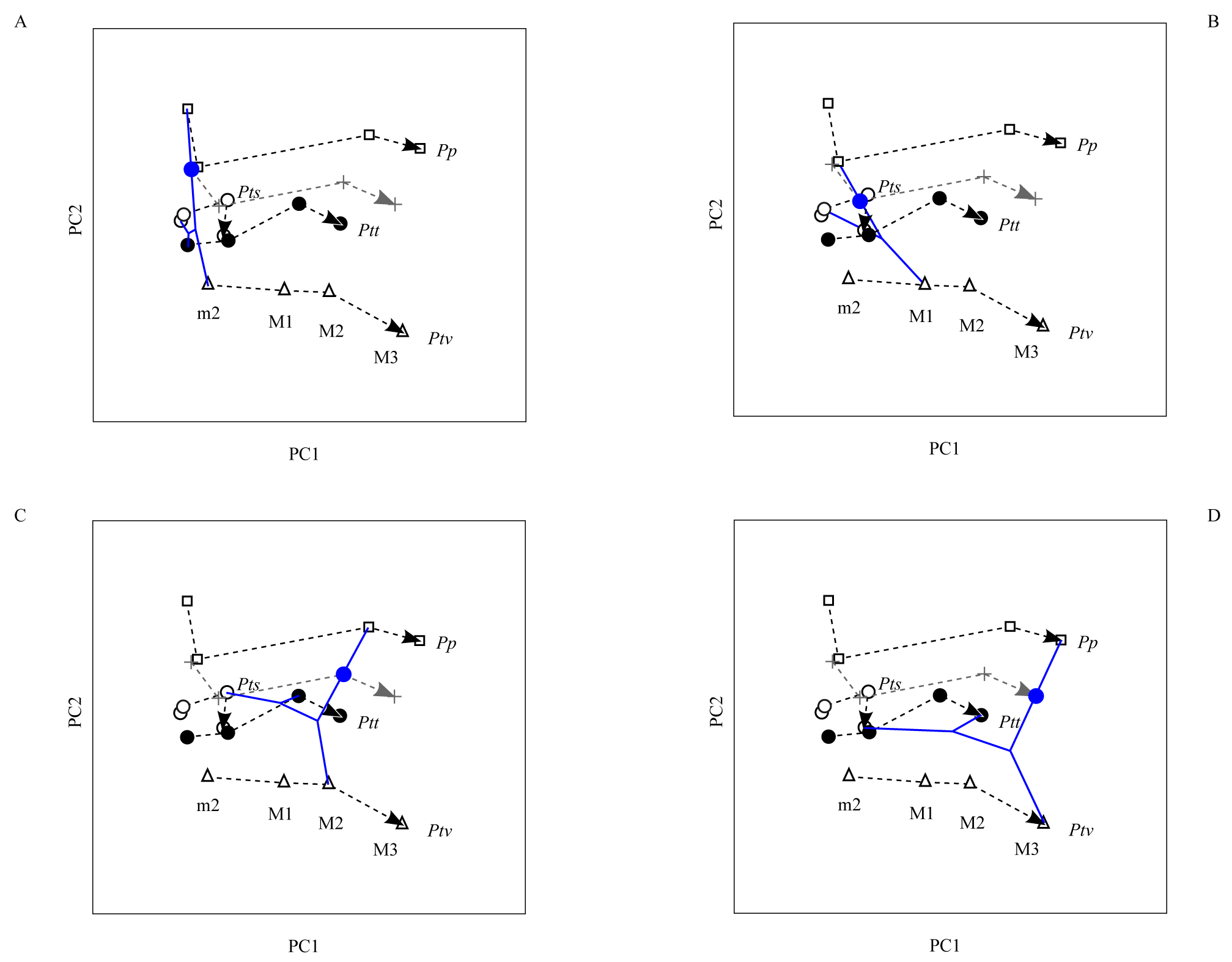

Supplement: Figure S4 — Phylogenetic tree in morphospace. The phylogenetic tree (blue lines; diamonds indicate the inferred state of last common ancestor at each ontogenetic stage) of the genus Pan is projected onto the shape space using a model of squared-change parsimony. A: m2 (infant), B: M1, C: M2, D: M3 (adult) stage. Gray symbols and line indicate the inferred ontogenetic trajectory of the last common ancestor. (TIF) [file pone.0102074.s004.tif]

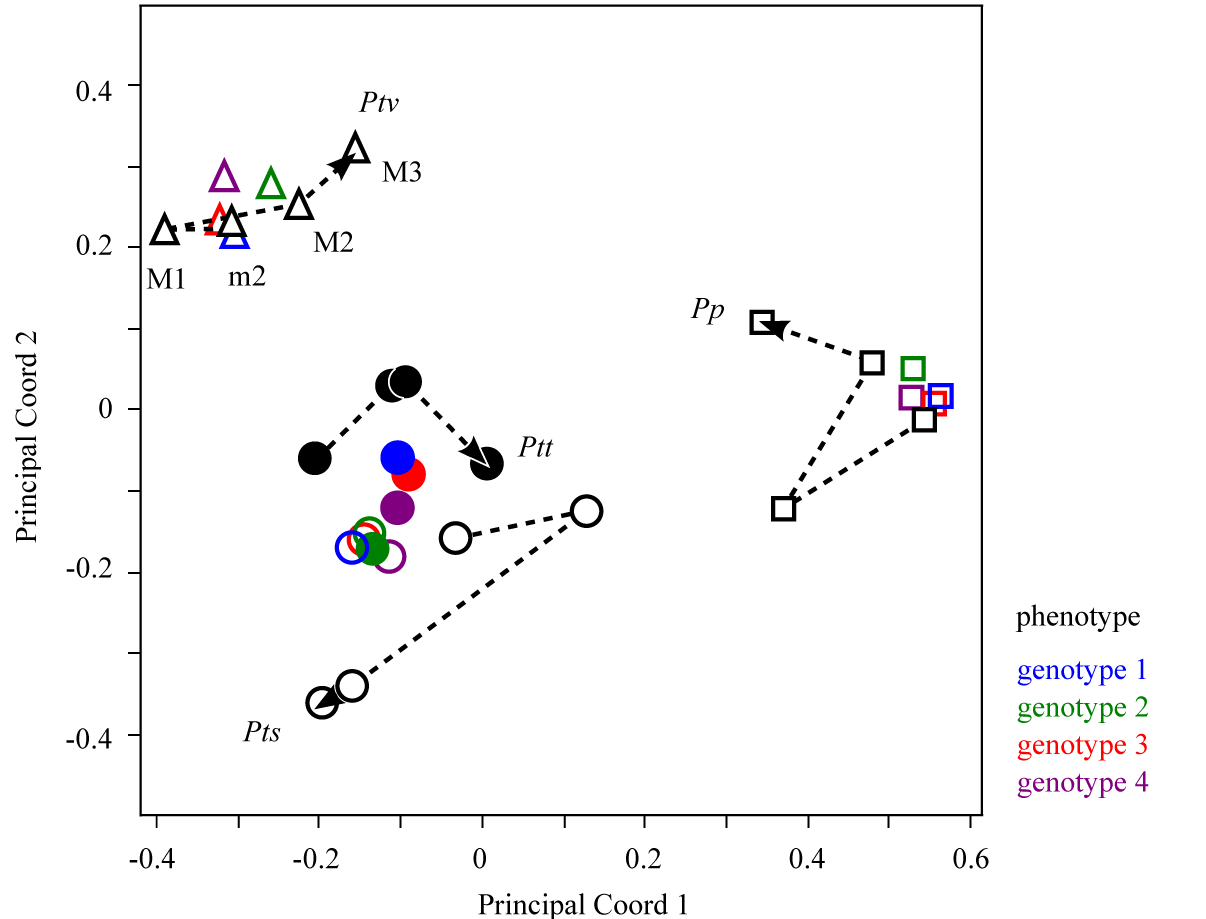

Supplement: Figure S5 — Phenetic and genetic similarity between Pan taxa. Principal Coordinates Analysis (PCO) of phenetic and genetic distance data. Phenetic data (black) are given for consecutive ontogenetic stages (connected with dashed lines). Genetic data (color) are from ref. [18] (blue), ref. [19] (green), ref. [21] (red), and ref. [22] (magenta). Note that during ontogeny the phenetic distance configuration departs from the genetic distance configuration. (TIF) [file pone.0102074.s005.tif]

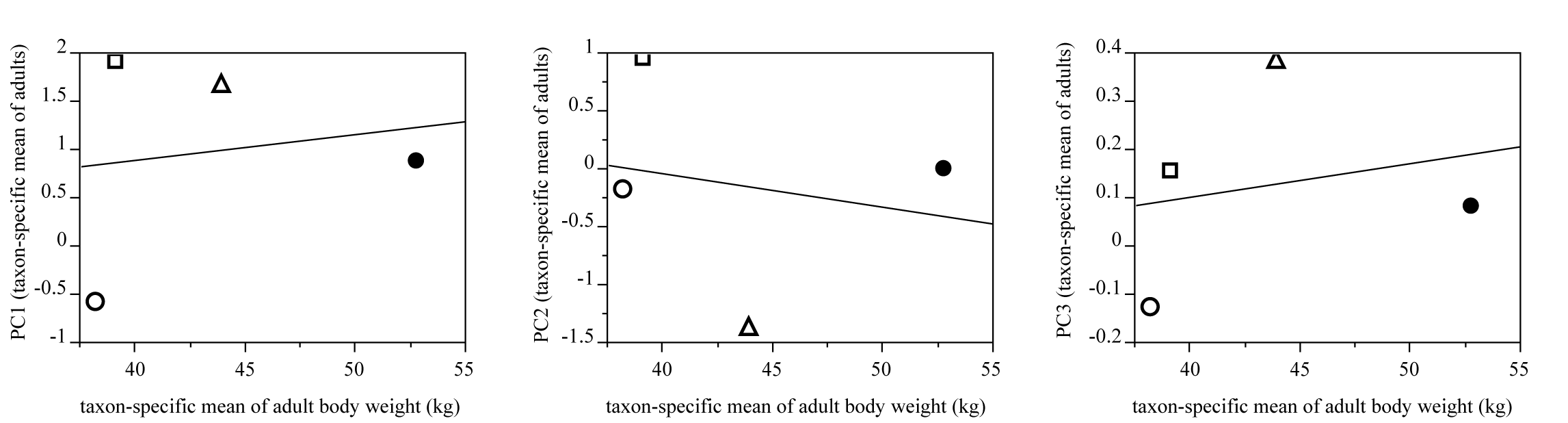

Supplement: Figure S6 — Correlation of taxon-specific means of adult body weight and PC scores. Taxon-specific means of adult body weight was calculated as a mean of male and female body weight taken from the literature [112]. (TIF) [file pone.0102074.s006.tif]
