# Supplementary material for: Phenotypic Variation in Infants, Not Adults, Reflects Genotypic Variation among Chimpanzees and Bonobos
Source: PLoS One. 2014 Jul 11;9(7):e102074. doi: 10.1371/journal.pone.0102074 (PMC4094530; doi:10.1371/journal.pone.0102074)
Supplement: Table S1 — Specimen list. The following specimens are used in this study. AIMUZH: Anthropological Institute and Museum of University of Zurich. MRA: Royal Africa Museum, Tervuren, Belgium. (DOCX) [file pone.0102074.s007.docx]

Table S1. Specimen list. The following specimens are used in this study. AIMUZH: Anthropological Institute and Museum of University of Zurich. MRA: Royal Africa Museum, Tervuren, Belgium.

| specimen ID | Taxon | Institution |
| --- | --- | --- |
| AIMUZH-AM7659 | *Pan troglodytes troglodytes* | AIMUZH |
| AIMUZH-AS1813 | *Pan troglodytes troglodytes* | AIMUZH |
| AIMUZH-AM8606 | *Pan troglodytes troglodytes* | AIMUZH |
| AIMUZH-AM6670 | *Pan troglodytes troglodytes* | AIMUZH |
| AIMUZH-AS1571 | *Pan troglodytes troglodytes* | AIMUZH |
| AIMUZH-PAL4 | *Pan troglodytes troglodytes* | AIMUZH |
| AIMUZH-AS1662 | *Pan troglodytes troglodytes* | AIMUZH |
| AIMUZH-AM10768 | *Pan troglodytes troglodytes* | AIMUZH |
| AIMUZH-AM10742 | *Pan troglodytes troglodytes* | AIMUZH |
| AIMUZH-AS1806 | *Pan troglodytes troglodytes* | AIMUZH |
| AIMUZH-AS1760 | *Pan troglodytes troglodytes* | AIMUZH |
| AIMUZH-AS788 | *Pan troglodytes troglodytes* | AIMUZH |
| AIMUZH-AS1742 | *Pan troglodytes troglodytes* | AIMUZH |
| AIMUZH-AM6695 | *Pan troglodytes troglodytes* | AIMUZH |
| AIMUZH-AS1787 | *Pan troglodytes troglodytes* | AIMUZH |
| AIMUZH-AM6613 | *Pan troglodytes troglodytes* | AIMUZH |
| AIMUZH-AM6614 | *Pan troglodytes troglodytes* | AIMUZH |
| AIMUZH-AS1786 | *Pan troglodytes troglodytes* | AIMUZH |
| AIMUZH-AM6615 | *Pan troglodytes troglodytes* | AIMUZH |
| AIMUZH-AM7480 | *Pan troglodytes troglodytes* | AIMUZH |
| AIMUZH-PAL221 | *Pan troglodytes troglodytes* | AIMUZH |
| AIMUZH-AM7421 | *Pan troglodytes troglodytes* | AIMUZH |
| AIMUZH-AS310 | *Pan troglodytes troglodytes* | AIMUZH |
| AIMUZH-AS1755 | *Pan troglodytes troglodytes* | AIMUZH |
| AIMUZH-AS1814 | *Pan troglodytes troglodytes* | AIMUZH |
| AIMUZH-AM7056 | *Pan troglodytes troglodytes* | AIMUZH |
| AIMUZH-AM7009 | *Pan troglodytes troglodytes* | AIMUZH |
| AIMUZH-AS1808 | *Pan troglodytes troglodytes* | AIMUZH |
| AIMUZH-AM6972 | *Pan troglodytes troglodytes* | AIMUZH |
| AIMUZH-PAL194 | *Pan troglodytes troglodytes* | AIMUZH |
| AIMUZH-AM6616 | *Pan troglodytes troglodytes* | AIMUZH |
| AIMUZH-PAL110 | *Pan troglodytes troglodytes* | AIMUZH |
| AIMUZH-AS1687 | *Pan troglodytes troglodytes* | AIMUZH |
| AIMUZH-PAL106 | *Pan troglodytes troglodytes* | AIMUZH |
| AIMUZH-AM11037 | *Pan troglodytes troglodytes* | AIMUZH |
| AIMUZH-AS1785 | *Pan troglodytes troglodytes* | AIMUZH |
| AIMUZH-AM6938 | *Pan troglodytes troglodytes* | AIMUZH |
| AIMUZH-AS1789 | *Pan troglodytes troglodytes* | AIMUZH |
| AIMUZH-AS1680 | *Pan troglodytes troglodytes* | AIMUZH |
| AIMUZH-PAL96 | *Pan troglodytes troglodytes* | AIMUZH |
| AIMUZH-AM7078 | *Pan troglodytes troglodytes* | AIMUZH |
| AIMUZH-PAL175 | *Pan troglodytes troglodytes* | AIMUZH |
| AIMUZH-AM6876 | *Pan troglodytes troglodytes* | AIMUZH |
| AIMUZH-AS1695 | *Pan troglodytes troglodytes* | AIMUZH |
| AIMUZH-AS1586 | *Pan troglodytes troglodytes* | AIMUZH |
| MRAC153 | *Pan troglodytes troglodytes* | MRA |
| MRAC179 | *Pan troglodytes troglodytes* | MRA |
| MRAC3466 | *Pan troglodytes troglodytes* | MRA |
| MRAC15233 | *Pan troglodytes troglodytes* | MRA |
| MRAC18188 | *Pan troglodytes troglodytes* | MRA |
| MRAC302 | *Pan troglodytes schweinfurthii* | MRA |
| MRAC9584 | *Pan troglodytes schweinfurthii* | MRA |
| MRAC10781 | *Pan troglodytes schweinfurthii* | MRA |
| MRAC13759 | *Pan troglodytes schweinfurthii* | MRA |
| MRAC21700 | *Pan troglodytes schweinfurthii* | MRA |
| MRAC23501 | *Pan troglodytes schweinfurthii* | MRA |
| MRAC23502 | *Pan troglodytes schweinfurthii* | MRA |
| MRAC23503 | *Pan troglodytes schweinfurthii* | MRA |
| MRAC23504 | *Pan troglodytes schweinfurthii* | MRA |
| MRAC26493 | *Pan troglodytes schweinfurthii* | MRA |
| MRAC5894 | *Pan troglodytes schweinfurthii* | MRA |
| MRAC11527 | *Pan troglodytes schweinfurthii* | MRA |
| MRAC12185 | *Pan troglodytes schweinfurthii* | MRA |
| MRAC12231 | *Pan troglodytes schweinfurthii* | MRA |
| MRAC13094 | *Pan troglodytes schweinfurthii* | MRA |
| MRAC13717 | *Pan troglodytes schweinfurthii* | MRA |
| MRAC23506 | *Pan troglodytes schweinfurthii* | MRA |
| MRAC25491 | *Pan troglodytes schweinfurthii* | MRA |
| MRAC29076 | *Pan troglodytes schweinfurthii* | MRA |
| MRAC458 | *Pan troglodytes schweinfurthii* | MRA |
| MRAC2487 | *Pan troglodytes schweinfurthii* | MRA |
| MRAC5378 | *Pan troglodytes schweinfurthii* | MRA |
| MRAC5893 | *Pan troglodytes schweinfurthii* | MRA |
| MRAC13716 | *Pan troglodytes schweinfurthii* | MRA |
| MRAC23505 | *Pan troglodytes schweinfurthii* | MRA |
| MRAC29077 | *Pan troglodytes schweinfurthii* | MRA |
| MRAC30676 | *Pan troglodytes schweinfurthii* | MRA |
| MRAC1048 | *Pan troglodytes schweinfurthii* | MRA |
| MRAC2298 | *Pan troglodytes schweinfurthii* | MRA |
| MRAC4188 | *Pan troglodytes schweinfurthii* | MRA |
| MRAC5891 | *Pan troglodytes schweinfurthii* | MRA |
| MRAC5892 | *Pan troglodytes schweinfurthii* | MRA |
| MRAC7004 | *Pan troglodytes schweinfurthii* | MRA |
| MRAC11362 | *Pan troglodytes schweinfurthii* | MRA |
| MRAC11363 | *Pan troglodytes schweinfurthii* | MRA |
| MRAC15350 | *Pan troglodytes schweinfurthii* | MRA |
| MRAC19534 | *Pan troglodytes schweinfurthii* | MRA |
| MRAC29074 | *Pan troglodytes schweinfurthii* | MRA |
| MRAC29078 | *Pan troglodytes schweinfurthii* | MRA |
| AIMUZH-AM13432 | *Pan troglodytes verus* | AIMUZH |
| AIMUZH-AM11777 | *Pan troglodytes verus* | AIMUZH |
| AIMUZH-AM11788 | *Pan troglodytes verus* | AIMUZH |
| AIMUZH-AM14993 | *Pan troglodytes verus* | AIMUZH |
| AIMUZH-AM14995 | *Pan troglodytes verus* | AIMUZH |
| AIMUZH-AM14992 | *Pan troglodytes verus* | AIMUZH |
| AIMUZH-AM13433 | *Pan troglodytes verus* | AIMUZH |
| AIMUZH-AM12175 | *Pan troglodytes verus* | AIMUZH |
| AIMUZH-AM11791 | *Pan troglodytes verus* | AIMUZH |
| AIMUZH-AM11776 | *Pan troglodytes verus* | AIMUZH |
| AIMUZH-AM13437 | *Pan troglodytes verus* | AIMUZH |
| AIMUZH-AM11792 | *Pan troglodytes verus* | AIMUZH |
| AIMUZH-AM11779 | *Pan troglodytes verus* | AIMUZH |
| AIMUZH-AM11778 | *Pan troglodytes verus* | AIMUZH |
| AIMUZH-AM11800 | *Pan troglodytes verus* | AIMUZH |
| AIMUZH-AM11785 | *Pan troglodytes verus* | AIMUZH |
| AIMUZH-AM11781 | *Pan troglodytes verus* | AIMUZH |
| AIMUZH-AM11786 | *Pan troglodytes verus* | AIMUZH |
| AIMUZH-AM11775 | *Pan troglodytes verus* | AIMUZH |
| AIMUZH-AM11903 | *Pan troglodytes verus* | AIMUZH |
| AIMUZH-AM13429 | *Pan troglodytes verus* | AIMUZH |
| AIMUZH-AM11780 | *Pan troglodytes verus* | AIMUZH |
| AIMUZH-AM13438 | *Pan troglodytes verus* | AIMUZH |
| AIMUZH-AM13439 | *Pan troglodytes verus* | AIMUZH |
| AIMUZH-AM14994 | *Pan troglodytes verus* | AIMUZH |
| AIMUZH-AM14996 | *Pan troglodytes verus* | AIMUZH |
| MRAC11293 | *Pan paniscus* | MRA |
| MRAC22336 | *Pan paniscus* | MRA |
| MRAC84036 M05 | *Pan paniscus* | MRA |
| MRAC29012 | *Pan paniscus* | MRA |
| AIMUZH-AS1768 | *Pan paniscus* | AIMUZH |
| MRAC23464 | *Pan paniscus* | MRA |
| MRAC12087 | *Pan paniscus* | MRA |
| MRAC22907 | *Pan paniscus* | MRA |
| MRAC11528 | *Pan paniscus* | MRA |
| MRAC29028 | *Pan paniscus* | MRA |
| MRAC29056 | *Pan paniscus* | MRA |
| MRAC29048 | *Pan paniscus* | MRA |
| MRAC22908 | *Pan paniscus* | MRA |
| MRAC29058 | *Pan paniscus* | MRA |
| MRAC5374 | *Pan paniscus* | MRA |
| MRAC29032 | *Pan paniscus* | MRA |
| MRAC84036 M03 | *Pan paniscus* | MRA |
| MRAC29057 | *Pan paniscus* | MRA |
| MRAC29055 | *Pan paniscus* | MRA |
| MRAC29053 | *Pan paniscus* | MRA |
| MRAC29054 | *Pan paniscus* | MRA |
| MRAC29036 | *Pan paniscus* | MRA |
| MRAC23509 | *Pan paniscus* | MRA |
| MRAC13201 | *Pan paniscus* | MRA |
| MRAC15295 | *Pan paniscus* | MRA |
| MRAC27696 | *Pan paniscus* | MRA |
| MRAC29045 | *Pan paniscus* | MRA |
| MRAC15294 | *Pan paniscus* | MRA |
| MRAC15296 | *Pan paniscus* | MRA |
| MRAC15293 | *Pan paniscus* | MRA |
| MRAC29040 | *Pan paniscus* | MRA |
