# Supplementary material for: Phenotypic Variation in Infants, Not Adults, Reflects Genotypic Variation among Chimpanzees and Bonobos
Source: PLoS One. 2014 Jul 11;9(7):e102074. doi: 10.1371/journal.pone.0102074 (PMC4094530; doi:10.1371/journal.pone.0102074)
Supplement: Table S3 — Phenetic distances between taxon-specific mean shapes. (DOCX) [file pone.0102074.s009.docx]

Table S3. Phenetic distances between taxon-specific mean shapes

| Ontogeny mean | *P. t. t.* | *P. t. s.* | *P. t. v.* |
| --- | --- | --- | --- |
| *P. t. s.* | 0.67* |  |  |
| *P. t. v.* | 1.48* | 2.10* |  |
| *P. p.* | 1.37* | 1.75* | 2.13* |

| m2 | *P. t. t.* | *P. t. s.* | *P. t. v.* |
| --- | --- | --- | --- |
| *P. t. s.* | 0.39 |  |  |
| *P. t. v.* | 0.56 | 0.93 |  |
| *P. p.* | 1.75* | 1.48* | 2.23* |

| M1 | *P. t. t.* | *P. t. s.* | *P. t. v.* |
| --- | --- | --- | --- |
| *P. t. s.* | 0.71 |  |  |
| *P. t. v.* | 1.04 | 1.75* |  |
| *P. p.* | 1.01* | 0.71 | 1.93* |

| M2 | *P. t. t.* | *P. t. s.* | *P. t. v.* |
| --- | --- | --- | --- |
| *P. t. s.* | 0.93 |  |  |
| *P. t. v.* | 1.18 | 1.78* |  |
| *P. p.* | 1.41* | 2.01* | 2.18* |

| M3 | *P. t. t.* | *P. t. s.* | *P. t. v.* |
| --- | --- | --- | --- |
| *P. t. s.* | 1.49* |  |  |
| *P. t. v.* | 1.62* | 2.61* |  |
| *P. p.* | 1.41* | 2.75* | 2.34* |

**p*<0.05
