# Supplementary material for: Phenotypic Variation in Infants, Not Adults, Reflects Genotypic Variation among Chimpanzees and Bonobos
Source: PLoS One. 2014 Jul 11;9(7):e102074. doi: 10.1371/journal.pone.0102074 (PMC4094530; doi:10.1371/journal.pone.0102074)
Supplement: Table S4 — Divergence of ontogenetic vector. (DOCX) [file pone.0102074.s010.docx]

Table S4. Divergence of ontogenetic vector

|  | *P. t. t.* | *P. t. s.* | *P. t. v.* |
| --- | --- | --- | --- |
| *P. t. s.* | 0.011 (*p*=0.95) |  |  |
| *P. t. v.* | 0.112 (*p*=0.39) | 0.071 (*p*=0.79) |  |
| *P. p.* | 0.054 (*p*=0.50) | 0.027 (*p*=0.88) | 0.011 (*p*=0.87) |
