# Supplementary material for: Phenotypic Variation in Infants, Not Adults, Reflects Genotypic Variation among Chimpanzees and Bonobos
Source: PLoS One. 2014 Jul 11;9(7):e102074. doi: 10.1371/journal.pone.0102074 (PMC4094530; doi:10.1371/journal.pone.0102074)
Supplement: Table S5 — F-test on taxon-specific variance along PC1. (DOCX) [file pone.0102074.s011.docx]

Table S5. F-test on taxon-specific variance along PC1

|  | *P. t. t.* | *P. t. s.* | *P. t. v.* |
| --- | --- | --- | --- |
| *P. t. s.* | *P.t.t.* > *P.t.s.* * |  |  |
| *P. t. v.* | *p*=0.494 | *P.t.v.* > *P.t.s.* * | |
| *P. p.* | *P.p.* > *P.t.t.* * | *P.p.* > *P.t.s.* * | *p*=0.164 |

**p*<0.05
