# Supplementary material for: Phenotypic Variation in Infants, Not Adults, Reflects Genotypic Variation among Chimpanzees and Bonobos
Source: PLoS One. 2014 Jul 11;9(7):e102074. doi: 10.1371/journal.pone.0102074 (PMC4094530; doi:10.1371/journal.pone.0102074)
Supplement: Table S6 — Correlation of genetic and phenetic distances. (DOCX) [file pone.0102074.s012.docx]

Table S6. Correlation of genetic and phenetic distances

|  | *D*_a_ |  | *D*_CH_ |  | *D*_PPC_ |  | *F*_ST_^5^ |  |
| --- | --- | --- | --- | --- | --- | --- | --- | --- |
|  | *r* | *p* | *r* | *p* | *r* | *p* | *r* | *p* |
| *D*_a_ ^1^ | - | - |  |  |  |  |  |  |
| *D*_CH_ ^2^ | 0.98 | 0.01 | - | - |  |  |  |  |
| *D*_PPC_ ^3^ | 0.97 | <0.01 | 0.99 | <0.01 | - | - |  |  |
| *F*_ST_ | 0.96 | <0.01 | 0.97 | <0.01 | 0.99 | <0.01 | - | - |
| *D*_ph_ ^4^ | 0.95 | <0.01 | 0.91 | <0.01 | 0.94 | <0.01 | 0.92 | <0.01 |

Correlation (*r*) and significance levels (*p*) are evaluated with Mantel test (1000 permutations)

^1^ Nei's standard distance

^2^ Cavalli-Sforze and Edward chord distance

^3^ Euclidean distance in Patterson's PC space

^4^ Euclidean distance in morphospace (shape PCs) (m2: infant stage)

^5^ Fisher et al., 2011
