# Supplementary material for: Phenotypic Variation in Infants, Not Adults, Reflects Genotypic Variation among Chimpanzees and Bonobos
Source: PLoS One. 2014 Jul 11;9(7):e102074. doi: 10.1371/journal.pone.0102074 (PMC4094530; doi:10.1371/journal.pone.0102074)
Supplement: Table S7 — Correlation between phenetic and genetic distance matrices. (DOCX) [file pone.0102074.s013.docx]

Table S7. Correlation between phenetic and genetic distance matrices^1^

|  | phenotype m2 | | phenotype M1 | | phenotype M2 | | phenotype M3 | |
| --- | --- | --- | --- | --- | --- | --- | --- | --- |
|  | *r* | *p* | *r* | *p* | *r* | *p* | *r* | *p* |
| genotype 1 (ref. [[1](#_ENREF_1)]) | 0.95 | **0.0029** | 0.50 | 0.1431 | 0.82 | **0.0316** | 0.46 | 0.1624 |
| genotype 2 (ref. [[2](#_ENREF_2)]) | 0.91 | **0.0046** | 0.48 | 0.1641 | 0.69 | 0.0945 | 0.42 | 0.1890 |
| genotype 3 (ref. [[3](#_ENREF_3)]) | 0.95 | **0.0044** | 0.54 | 0.1415 | 0.79 | **0.0270** | 0.44 | 0.1756 |
| genotype 4 (ref. [[4](#_ENREF_4)]) | 0.92 | **0.0034** | 0.60 | 0.1014 | 0.72 | 0.0619 | 0.47 | 0.1730 |

^1^: correlation (*r*) and significance levels (*p*) evaluated with Mantel test (10,000 permutations)

**References**

1. Becquet C, Patterson N, Stone AC, Przeworski M, Reich D (2007) Genetic structure of chimpanzee populations. PLoS Genetics 3: e66.

2. Gonder MK, Locatelli S, Ghobrial L, Mitchell MW, Kujawski JT, et al. (2011) Evidence from Cameroon reveals differences in the genetic structure and histories of chimpanzee populations. Proceedings of the National Academy of Sciences of the United States of America 108: 4766-4771.

3. Fischer A, Pollack J, Thalmann O, Nickel B, Pääbo S (2006) Demographic history and genetic differentiation in apes. Current Biology 16: 1133-1138.

4. Fischer A, Prufer K, Good JM, Halbwax M, Wiebe V, et al. (2011) Bonobos fall within the genomic variation of chimpanzees. PLoS ONE 6: e21605.
