# Supplementary material for: Phenotypic Variation in Infants, Not Adults, Reflects Genotypic Variation among Chimpanzees and Bonobos
Source: PLoS One. 2014 Jul 11;9(7):e102074. doi: 10.1371/journal.pone.0102074 (PMC4094530; doi:10.1371/journal.pone.0102074)
Supplement: Table S8 — Correlation between PC scores and taxon-specific adult body masses. (DOCX) [file pone.0102074.s014.docx]

Table S8. Correlation between PC scores and taxon-specific adult body masses

| PC1 |  | PC2 |  | PC3 |  |
| --- | --- | --- | --- | --- | --- |
| *R*­­^2^ | *p* | *R*­­^2^ | *p* | *R*­­^2^ | *p* |
| 0.03 | 0.84 | 0.04 | 0.80 | 0.05 | 0.78 |
