# Supplementary material for: Phenotypic Variation in Infants, Not Adults, Reflects Genotypic Variation among Chimpanzees and Bonobos
Source: PLoS One. 2014 Jul 11;9(7):e102074. doi: 10.1371/journal.pone.0102074 (PMC4094530; doi:10.1371/journal.pone.0102074)
Supplement: Text S1 — Materials and methods. (DOCX) [file pone.0102074.s015.docx]

**Text S1: Materials and methods**

**Sample structure.** Postnatal ontogenetic stages were defined as follows: stage 1 (infant): second milk molar (m2) erupted; stage 2: first permanent molar (M1) erupted , stage 3: M2 erupted, stage 4 (adult): M3 erupted. The sample consists of femora of *Pan troglodytes troglodytes* (*N*=50; *N*=12, 18, 9, 11 for m2, M1/2/3 eruption respectively]), *Pan t. schweinfurthii* (*N*=39; *N*=10, 9, 8, 12 for each stage), *Pan t. verus* (*N*=26; *N*=4, 4, 5, 13 for each stage) and *Pan paniscus* (bonobo) (*N*=31; *N*= 4, 8, 10, 9 for each stage) (Fig. S2; pooled sex). Femoral diaphyseal length in this sample varies between 67mm and 227mm). Most specimens are wild-shot except for *P.t.t.* (wild: *N*=23, captive: *N*=19, status unknown: *N*=8). *P. t. troglodytes* and *P. t. verus* specimens were obtained from the collections of the Anthropological Institute and Museum of the University of Zurich (AIMUZH), *P. t. schweifurthii* specimens were obtained from the collections of the Royal Africa Museum, Tervuren, Belgium (MRA), and *P. paniscus* specimens were obtained from AIMUZH and MRA (Table S1).

**Volumetric data acquisition.** Femora were scanned using a Siemens 64-detector-array CT device (beam collimation 1.0mm; standard/bone reconstruction kernels [B30/B60]; interslice distance between 0.2 and 0.5mm). Small specimens were scanned using a micro-CT scanner (µCT80, Scanco Medical, Switzerland; volume data reconstructed at an isotropic voxel resolution of 75 µm). Cross-sections orthogonal to the principal axis of the femoral shaft were obtained by resampling the original volumetric data using the software Amira 4.1 (Mercury Systems).

**Morphometric data acquisition.** In immature specimens, unfused epiphyses are often missing, or their position relative to the diaphysis cannot be reconstructed reliably. We thus focus here on diaphyseal morphology. The femoral diaphysis was extracted from the CT volume data using epiphyseal lines as proximal and distal delimiters. Subperiosteal (external) outlines of each cross section were parameterized with elliptical Fourier analysis (EFA) [[1](#_ENREF_1)]. EFA was used to reduce noise, and to define parametric outline functions. The curvature of the external diaphyseal surface *k_ext_* was calculated analytically using the parametric functions of EFA. Resulting positive/negative values of the curvature *k_ext_* denote convex/concave regions, respectively (see ref. [[2](#_ENREF_2)] for details).

**Morphometric analysis.** For each specimen, measurements of *k_ext_* were sampled around each cross-sectional outline, and along the entire diaphyseal shaft. These data were normalized to their respective median values, and mapped onto a cylindrical coordinate system (*ρ*, *θ*, *z*), where *ρ*=1/(2π)=const. denotes the radius of the cylinder, angle *θ* denotes the anatomical direction (*θ*=0º→360º: anterior → medial → posterior → lateral → anterior), and *z* denotes the normalized position along the diaphysis (*z*=0 → 1: distal → proximal) [[3](#_ENREF_3),[4](#_ENREF_4)]. Since *ρ*=const., data can be visualized as two-dimensional morphometric maps **M**(*θ*, *z*), and distributions *k_ext_*(*θ*, *z*) (Fig. S3, Fig. 2) can be represented as *K*×*L* matrices, where *K* and *L* denote the number of elements along *z* and *θ*, respectively (*K*=*L*=300).

For the comparative analysis of the morphometric maps **M**, 2D-Fourier transforms *F*(**M**) were calculated (**M** has a natural periodicity in *θ*), yielding *K*×*L* sets of Fourier coefficients. Note that Fourier transform is applied to the size-corrected data. Specimens were aligned to each other by minimizing inter-specimen distances in Fourier space through rotation around *θ* (diaphyseal axis). To identify principal patterns of shape variation in the sample, Fourier coefficient sets were submitted to Principal Components Analysis (PCA). To facilitate visual inspection and anatomical interpretation of the results of PCA, real-space morphometric maps were reconstructed by transforming a given point **P*** in PC space into its corresponding set of Fourier coefficients *F*(**M***), and applying an inverse Fourier transform to obtain a morphometric map **M***. Morphometric maps were false-color coded.

Trajectory divergence *V_ij_* was calculated as

$V_{ij}=1-\left(a_{i}\cdot a_{j}\right)^{2}$,

where **a***_i_* and **a***_j_* are normalized trajectory direction vectors (multivariate regression of shape against diaphyseal length [[5](#_ENREF_5),[6](#_ENREF_6)]). Larger value of *V_ij_* means larger divergence between two vectors. All calculations were performed with MATLAB7.7 (MathWorks).

**Genetic distances.** Prior to the analyses, positions with a minor allele frequency (<5%) were removed. Inter-taxon genetic distances were evaluated by Nei’s standard distance [[7](#_ENREF_7)] and Cavalli-Sforze and Edwards chord distance [[8](#_ENREF_8)] based on taxon-specific allele frequencies. Principal component analysis (PCA) was performed on the coded SNPs depending on the number of variant allele [[9](#_ENREF_9)]. Inter-taxon distances were then evaluated as Euclidean distance between taxon-specific means in PC space. Inter-taxon Hamming distance [[10](#_ENREF_10)] was also calculated as the sum of Hamming distance of paternal/maternal strands. Since paternal/maternal attribution is unknown, we calculated the minimum Hamming distance.

**Resampling statistics.** To calculate the correlation between genetic and phenetic distances, we performed 1000 resampling operations for every pair of *Pan* taxa (i.e., six combinations), and for each ontogenetic stage. For each iteration *j* (*j*=1, … ,1000) on a pair of *X* (*x*_1_, *x*_2_, … , *x*_m_) and *Y* (*y*_1_, *y*_2_, … , *y*_n_) where *X* and *Y* denote the sample of each taxon, a pair of resampled specimens *X_j_*^*^(*x*_1_^*^ , *x*_2_^*^, …, *x*_m_^*^) and *Y_j_*^*^(*y*_1_^*^, *y*_2_^*^, …, *y*_n_^*^) were generated with replacement. Genetic distance Dg*_j_*^*^ was then calculated as Euclidean distance in Patterson’s PC space between mean points of specimens *X_j_*^*^ and *Y_j_*^*^ [[9](#_ENREF_9),[11](#_ENREF_11)] and as Nei’s standard distance [[7](#_ENREF_7)] from the allele frequencies in *X_j_*^*^ and *Y_j_*^*^. Likewise, but using different sets of specimens, phenetic distance Dp*_j_* ^*^ between every pair of *Pan* taxa was calculated in each iteration as Euclidean distance between taxon-specific means in PC space (*N*=3 dimensions). The genetic distance measures Dg^*^ and the phenetic distance measures Dp^*^ (both of which contain 1000×_4_C_2_=6000 distance measures) were normalized by standard deviation and plotted for each ontogenetic stage. See Fig. S3 too.

**Comparison of genetic and phenetic distance matrices.** To compare genetic and phenetic distances between taxa, a combination of Principal Coordinates Analysis (PCO) and Procrustes Analysis was used. PCO was applied to transform between-taxon distance matrices into matrices specifying the position of taxa relative to each other in multivariate space. The resulting genetic and phenetic “taxon constellations” were then superimposed with Procrustes Analysis to visualize the coincidence between genetic and phenetic distances. Correlation between phenetic and genetic distance data sets was assessed with the Mantel test. All calculations were performed with MATLAB7.7 (MathWorks). Split decomposition [[12](#_ENREF_12)] was applied to genetic and phenetic distance matrices using the software SplitsTree 4 [[13](#_ENREF_13)].

**References**

1. Kuhl F, Giardina C (1982) Elliptic Fourier features of a closed contour. Computer graphics and image processing 18: 236-258.

2. Morimoto N, Zollikofer CPE, Ponce de León MS (2011) Exploring femoral diaphyseal shape variation in wild and captive chimpanzees by means of morphometric mapping: a test of Wolff’s Law. Anatomical Record 294: 589-609.

3. Zollikofer CPE, Ponce de León MS (2001) Computer-assisted morphometry of hominoid fossils: the role of morphometric maps. In: De Bonis L, Koufos G, Andrews P, editors. Phylogeny of the Neogene Hominoid Primates of Eurasia. Cambridge: Cambridge University Press. pp. 50-59.

4. Bondioli L, Bayle P, Dean C, Mazurier A, Puymerail L, et al. (2010) Technical note: Morphometric maps of long bone shafts and dental roots for imaging topographic thickness variation. American Journal of Physical Anthropology 142: 328-334.

5. Zollikofer CPE, Ponce de León MS (2006) Neanderthals and modern humans - chimps and bonobos: similarities and differences in development and evolution. In: Harvati K, Harrison T, editors. Neanderthals Revisited: New Approaches and Perspectives. New York: Springer. pp. 71-88.

6. Penin X, Berge C, Baylac M (2002) Ontogenetic study of the skull in modern humans and the common chimpanzees: neotenic hypothesis reconsidered with a tridimensional Procrustes analysis. Am J Phys Anthropol 118: 50-62.

7. Nei M, Tajima F, Tateno Y (1983) Accuracy of estimated phylogenetic trees from molecular data. II. Gene frequency data. Journal of Molecular Evolution 19: 153-170.

8. Cavalli-Sforza LL, Edwards AW (1967) Phylogenetic analysis. Models and estimation procedures. Am J Hum Genet 19: 233-257.

9. Patterson N, Price AL, Reich D (2006) Population structure and eigenanalysis. PLoS Genetics 2: e190.

10. Hamming RW (1950) Error detecting and error correcting codes. The Bell System Technical Journal 29: 147-160.

11. Price AL, Patterson NJ, Plenge RM, Weinblatt ME, Shadick NA, et al. (2006) Principal components analysis corrects for stratification in genome-wide association studies. Nat Genet 38: 904-909.

12. Bandelt H-J, Dress AWM (1992) Split decomposition: A new and useful approach to phylogenetic analysis of distance data. Molecular Phylogenetics and Evolution 1: 242-252.

13. Huson DH, Bryant D (2006) Application of phylogenetic networks in evolutionary studies. Molecular Biology and Evolution 23: 254-267.
