# Supplementary material for: Phenotypic Variation in Infants, Not Adults, Reflects Genotypic Variation among Chimpanzees and Bonobos
Source: PLoS One. 2014 Jul 11;9(7):e102074. doi: 10.1371/journal.pone.0102074 (PMC4094530; doi:10.1371/journal.pone.0102074)
Supplement: Text S2 — Habitats of Pan taxa. (DOCX) [file pone.0102074.s016.docx]

**Text S2: Habitats of *Pan* taxa**

*Pan* species and subspecies have a relatively wide geographical distribution. *P. troglodytes schweinfurthii* occupies closed forest woodland (Mahale), and thicket woodland and semideciduous forest (Gombe). *P. t. verus* occupies lowland evergreen rainforest. *P. paniscus* occupies primary lowland rainforest [[1](#_ENREF_1)]. It has been reported that the *Pan troglodytes troglodytes* habitat is different from habitats of other *Pan* taxa in terms of the forest composition. The habitat of *Pan troglodytes troglodytes* consists of a mosaic of coastal forest, patches of savanna, swamps, and mature forest (Loango) [[2](#_ENREF_2),[3](#_ENREF_3),[4](#_ENREF_4)].

**References**

1. Doran DM (1996) Comparative positional behabior of the African apes. In: McGrew MC, Marchant LF, Nishida T, editors. Great Ape Societies. Cambridge: Cambridge University Press.

2. Head J, Boesch C, Makaga L, Robbins M (2011) Sympatric chimpanzees (*Pan troglodytes troglodytes*) and gorillas (*Gorilla gorilla gorilla*) in Loango National Park, Gabon: Dietary fomposition, seasonality, and intersite comparisons. International Journal of Primatology 32: 755-775.

3. Oelze VM, Head JS, Robbins MM, Richards M, Boesch C (2014) Niche differentiation and dietary seasonality among sympatric gorillas and chimpanzees in Loango National Park (Gabon) revealed by stable isotope analysis. Journal of Human Evolution 66: 95-106.

4. Furuichi T, Inagaki H, Angoue-Ovono S (1997) Population density of chimpanzees and gorillas in the Petit Loango Reserve, Gabon: employing a new method to distinguish between nests of the two species. International Journal of Primatology 18: 1029-1046.
