# Supplementary material for: Phenotypic Variation in Infants, Not Adults, Reflects Genotypic Variation among Chimpanzees and Bonobos
Source: PLoS One. 2014 Jul 11;9(7):e102074. doi: 10.1371/journal.pone.0102074 (PMC4094530; doi:10.1371/journal.pone.0102074)
Supplement: Text S3 — In-vivo bone modification in the femur of Pan taxa. (DOCX) [file pone.0102074.s017.docx]

**Text S3: *In-vivo* bone modification in the femur of *Pan* taxa**

There are two basic factors governing variation in femoral morphology. One is short-term functional (environmental) modification during an individual’s life time, (*in-vivo* modification [„Wolff’s Law“ [[1](#_ENREF_1)]]), the other is long-term change due to neutral and/or adaptive evolution. In *Pan* taxa, effects of *in-vivo* bone modification have been tested in populations of *P. t. troglodytes*, *P. t. schweinfurthii*, and *P. t. verus*. Surprisingly, correlations between individual locomotor behaviors and *in-vivo* modification of femoral diaphyseal morphology seem to be weak to absent in the populations that have been studied so far [[2](#_ENREF_2),[3](#_ENREF_3),[4](#_ENREF_4)]. A study comparing the cross-sectional properties of femoral/humeral diaphysis in *P. t. verus* and *P. t. schweinfurthii* has found only loose correlation with population-specific locomotor behaviors [[2](#_ENREF_2),[5](#_ENREF_5)]. On the other hand, there is growing evidence that taxon-specific developmental programs play a central role to determine the morphology of the locomotor system [[4](#_ENREF_4)]. *Pan* exhibits a taxon-specific femoral morphology distinct from humans and other great ape species already at birth [[6](#_ENREF_6)]. Ontogenetic changes in femoral morphology reflect an underlying developmental program that is fairly independent of environmental influences [[3](#_ENREF_3),[4](#_ENREF_4)], and there is a clear effect of aging during postnatal ontogeny [[3](#_ENREF_3),[4](#_ENREF_4)]. Overall, these studies indicate that gene-mediated taxon-specific developmental programs are the dominant factor bringing about variation of femoral morphology within and between *Pan* taxa. The question that remains open is to which extent neutral versus non-neutral processes contributed to the evolution of taxon-specific femoral ontogeny and morphology in *Pan*.

**References**

1. Wolff J (1892) Das Gesetz der Transformation der Knochen. Berlin: A. Hirschwald.

2. Carlson KJ, Doran-Sheehy DM, Hunt KD, Nishida T, Yamanaka A, et al. (2006) Locomotor behavior and long bone morphology in individual free-ranging chimpanzees. Journal of Human Evolution 50: 394-404.

3. Carlson K, Sumner D, Morbeck M, Nishida T, Yamanaka A, et al. (2008) Role of nonbehavioral factors in adjusting long bone siaphyseal atructure in free-ranging *Pan troglodytes*. International Journal of Primatology 29: 1401-1420.

4. Morimoto N, Zollikofer CPE, Ponce de León MS (2011) Exploring femoral diaphyseal shape variation in wild and captive chimpanzees by means of morphometric mapping: a test of Wolff’s Law. Anatomical Record 294: 589-609.

5. Carlson KJ (2005) Investigating the form-function interface in African apes: Relationships between principal moments of area and positional behaviors in femoral and humeral diaphyses. Am J Phys Anthropol 127: 312-334.

6. Morimoto N, Zollikofer CPE, Ponce de León MS (2012) Shared human-chimpanzee pattern of perinatal femoral shaft morphology and its implications for the evolution of hominin locomotor adaptations. PLoS ONE 7: e41980.
